# Co‐Developing User‐Centred Nutrition Educational Resources to Integrate Nutrition Into Multiple Sclerosis Care: A Collaborative Approach With Healthcare Professionals and Healthcare Consumers

**DOI:** 10.1111/hex.70656

**Published:** 2026-04-02

**Authors:** Shoroog Allogmanny, Yasmine Probst, Karen Zoszak, Anita Stefoska‐Needham

**Affiliations:** ^1^ School of Medical, Indigenous and Health Sciences, Faculty of Science Medicine and Health, University of Wollongong Wollongong NSW Australia; ^2^ Clinical Nutrition Department, College of Applied Medical Sciences Taibah University Madinah Kingdom of Saudi Arabia; ^3^ School of Health Sciences, Faculty of Medicine and Health University of New South Wales Sydney NSW Australia

**Keywords:** co‐develop, educational resource, healthcare consumer, healthcare professional, multiple sclerosis, nutrition

## Abstract

**Introduction:**

Nutrition is often overlooked in multiple sclerosis (MS) care, leading to a gap in comprehensive patient management. Developing MS‐specific nutrition educational resources could address this gap. This study therefore aimed to collaboratively develop MS‐specific nutrition educational resources with healthcare professionals (HCPs) and people living with MS (plwMS) to support the future integration of nutrition into MS care.

**Methods:**

A three‐phased qualitative study was conducted, guided by the Design Thinking framework. Phase 1 involved separate online workshops with HCPs and plwMS, alongside semi‐structured interviews with HCPs, to explore needs and challenges related to nutrition in MS care, and to generate solution‐focused ideas for resource development. Phase 2 focused on developing prototypes. Phase 3 included usability testing of the co‐developed prototypes through online and face‐to‐face interviews with HCPs and plwMS. Data from Phases 1 and 3 were analysed using an inductive, reflexive thematic analysis.

**Results:**

Two workshops (*n* = 9 HCPs; *n* = 14 plwMS) and ten HCP interviews were conducted in Phase 1, generating four themes: (1) Addressing healthcare barriers to providing nutrition education in MS care; (2) Supporting person‐centred nutrition communication; (3) Fostering equity through accessible and inclusive MS‐specific educational resources; and (4) The integral role of the dietitian in MS care. A set of new nutrition‐resource prototypes, informed by Phase 1 theme‐based design considerations, was co‐developed and hosted on a website (Phase 2). In Phase 3, 18 HCPs and 15 plwMS provided feedback on the prototypes, grouped across four themes: (1) Clear, targeted messaging; (2) Visually and informative design; (3) Lived experiences; and (4) Trust, credibility and connection, suggesting that the design of user‐centred educational resources can drive action, support decision‐making and enhance user trust. These insights informed resource refinements for colour, language clarity, content relevance and navigation options to support their adoption into MS care.

**Conclusion:**

This study co‐developed MS‐specific nutrition educational resources to integrate nutrition into MS care. Future research should evaluate their applicability and effectiveness in real‐world settings.

**Patient or Public Contribution:**

An MS consumer panel was consulted during the planning phase, and HCPs and plwMS contributed throughout the design process, including feedback on Phase 1 findings and prototypes.

## 1 Introduction

Multiple sclerosis (MS) is a neurodegenerative disease of the central nervous system, and there is growing interest in integrating pharmacologic therapy with lifestyle‐based approaches, including nutrition, to support disease management [1]. Current evidence‐based nutrition recommendations for people living with MS (plwMS) emphasise a balanced diet aligned with national dietary guidelines [2, 3]. Due to inconsistencies in the evidence supporting specific dietary approaches, MS‐specific nutritional guidelines are not currently available [2]. This has led to significant gaps in nutrition‐related resources for MS, which includes resources designed to support healthcare professionals (HCPs) in providing nutrition guidance [4]. Limited resource availability in turn contributes to a reported dissatisfaction among plwMS regarding their overall MS care experience [5, 6]. As a result, dietary self‐management has emerged as a global priority among plwMS [7, 8], with many advocating for the integration of reliable, evidence‐based nutrition guidance into routine care delivered by HCPs [5]. Addressing this call to action would require the development of MS‐specific nutrition educational resources that translate existing recommendations into practical tools to assist HCPs in embedding nutrition within their practice.

Educational resource development cannot occur in isolation; rather, it must be grounded in meaningful collaboration with those directly affected (i.e. plwMS and HCPs). The principle of ‘Nothing about us, without us’, endorsed by the World Health Organization's framework for integrated people‐centred health services [9], highlights the value of co‐development between HCPs and healthcare service users and advocates for designing *with* consumers, rather than designing *for* them [10]. The approach ensures that healthcare consumers (i.e., plwMS) and stakeholders (i.e., HCPs) collaborate as active partners in the design of prototypes and/or solutions, driven by a deep appreciation and understanding of their experiences, needs and ideas [11, 12, 13]. Embedding the voices of consumers and stakeholders from the outset may enhance the acceptability and real‐world translation of co‐designed prototypes into practice [11, 12], while ensuring that outcomes align with needs expressed directly by end users rather than assumptions made by designers and/or researchers. In recent years, collaborative approaches have been successfully applied in the nutritional management of cancer [14] and obesity [15], as evidenced by the development of nutrition educational tools, such as patient education materials (PEMs), to support HCPs in integrating evidence‐based nutrition into routine patient care.

A recent scoping review identified only two MS‐related educational resources designed for use by HCPs that involved HCPs and plwMS during their development; however, neither of these resources focused on nutrition [4]. This indicates a notable gap in the application of collaborative development of MS‐specific nutrition educational resources. Therefore, the aim of this study was to collaboratively develop MS‐specific nutrition educational resources to support the integration of nutrition into MS care. The objectives were to engage both HCPs and plwMS to: (1) explore needs and challenges regarding MS‐related nutrition care, and generate solution‐based ideas to inform the development of MS‐specific nutrition educational resources; (2) develop MS‐specific nutrition educational resource prototypes; and (3) test the usability of, and refine, the educational resource prototypes.

## 2 Materials and Methods

### 2.1 Study Design

**Figure 1:**
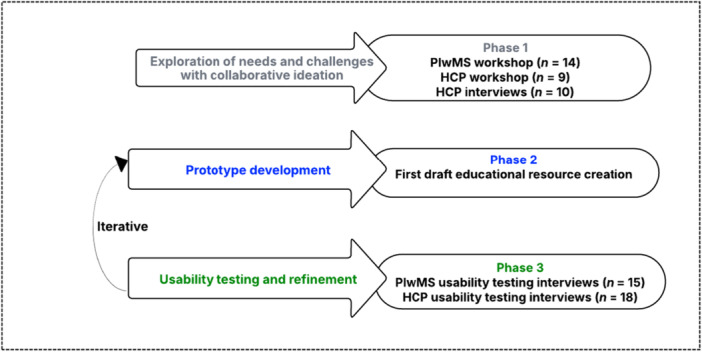
An overview of the study design and phases. HCPs, healthcare professionals; plwMS, people living with MS.

This qualitative study included workshops and semi‐structured interviews and was structured into three phases, aligned with the study objectives: (1) exploring needs and challenges, and generating solution‐based ideas, (2) developing MS‐specific nutrition educational resource prototypes, and (3) conducting usability testing and refining the prototypes (see Figure 1).

The constructivist paradigm guided the study design and data interpretation to comprehend how participants construct knowledge and make sense of their experiences regarding MS‐related nutrition care [16]. The study design was guided by key components of the Design Thinking framework (empathy, definition, ideation, prototyping and iterative testing) [17, 18], and incorporated a user‐centred design approach in which insights from HCPs and plwMS informed the development and refinement of the prototypes. The study was reported in accordance with the Consolidated Criteria for Reporting Qualitative Research (COREQ) guidelines for qualitative studies [19].

### 2.2 Phase 1: Exploration of Needs and Challenges With Collaborative Ideation

#### 2.2.1 Participant Sampling and Recruitment

Recruitment of a minimum of 20 key stakeholders, ten participants for the plwMS workshop and ten participants for the HCP workshop, was the target based on recommendations from previous research [20]. In this paper, the term ‘key stakeholders’ refers to both HCPs and plwMS. Inclusion criteria for both groups included (1) aged ≥ 18 years; (2) based in Australia; and (3) able to converse in the English language. HCPs were required to have MS‐related clinical or academic experience, while plwMS were required to have a self‐reported MS diagnosis, no severe cognitive impairment preventing communication, and not be pregnant or lactating. Recruitment commenced in August 2024.

Study invitations were sent to 22 plwMS and 42 HCPs who participated in our team's previous studies [5, 21] and had expressed willingness to engage in the present study. Additional key stakeholders were recruited via study advertisements on social media platforms and the MS Australia website. To ensure diversity, 20 HCPs with MS experience were invited through purposive sampling [22], using direct emails identified via open Google searches. Participants were also encouraged to share study information with relevant contacts [23]. Once participation was confirmed, a short questionnaire was emailed to collect sociodemographic data, as well as professional data from HCPs and clinical information from plwMS.

#### 2.2.2 Data Collection

Two separate, 2‐h online workshops were conducted via Zoom (version 5.9.1 https://zoom.us), one with plwMS (September 2024) and one with HCPs (October 2024). Separate workshops were held following consultation with an MS consumer group to create a supportive environment that enabled participants to openly share their experiences, challenges and needs. The workshops were planned in a hybrid format; however, the format was changed to online only due to a low interest for in‐person attendance. Seven days before the workshops, participants received a workshop agenda. Family members and/or carers were welcome to attend.

**Figure 2:**
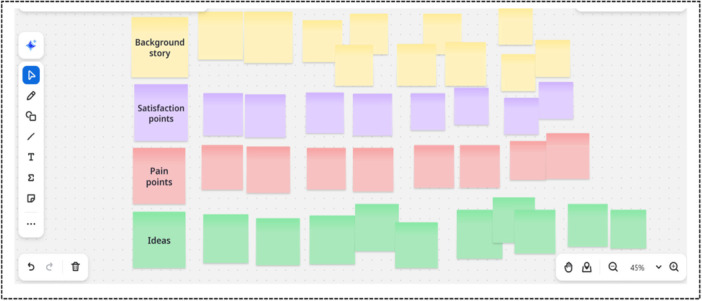
Screenshot of the digital whiteboard used in the workshops, sticky notes organised under four headings: background story; satisfaction points; pain points/challenges; and ideas (adapted from [26]).

Three facilitators (SA, KZ and ASN) led the plwMS workshop, and two facilitators (SA and KZ) led the HCP workshop. The workshop commenced with a 10‐min presentation outlining the project objectives to build empathy with the participants, guided by a previous design project [24]. The participants were then divided into small groups using online breakout rooms (three for plwMS, two for HCPs); each group consisted of four to five participants and was facilitated by one of the researchers (SA, KZ or ASN) to foster active engagement. In the HCP workshop, professionals from each discipline were intentionally grouped to mimic a multidisciplinary team in practice. Using a Design Thinking approach [18], facilitators guided the discussions within each group focusing on empathising with the end user (HCPs and plwMS) to explore their experiences, challenges, and needs related to MS‐related nutrition care. Facilitators also encouraged ideation of solutions using ‘*how might we…*’ focussed questions [25]. Key points were captured using digital sticky notes on the Zoom whiteboard under four headings: (1) background story; (2) satisfaction points; (3) pain points/challenges; and (4) ideas for change or solutions [26] (Figure 2). See Supporting Information 1 for additional context on these headings. The questions used to prompt the discussion were informed by insights from our previous studies (e.g., identified gaps in MS‐related nutrition educational resources and key stakeholder preferences) [4, 5, 21], as well as the research team's expertise. After the small group discussions, participants reconvened in the main Zoom session, where a representative from each group briefly summarised their group's whiteboard. The HCP workshop was recorded via Zoom, but only audio data was used for analysis. For the plwMS workshop, only the summary session was audio‐recorded using Otter AI (version 2.1.59.697 https://otter.ai) to foster a relaxed environment. Data included digital sticky notes, verbatim transcripts and saved whiteboards from each group for further analysis. After each workshop, the facilitators held a post‐workshop debrief.

The workshops were recognised as a challenging method for engaging HCPs due to time constraints [27]. In design‐led processes, flexible methods are commonly used to capture all stakeholders' perspectives [12, 28]. Individual interviews were, therefore, offered to HCPs who could not attend the workshop at a time convenient to them. All interviews were facilitated by the primary researcher (SA) and followed the same structure as the workshops to ensure consistency. Interviews were conducted and recorded via Zoom over a 4‐week period between October and November 2024.

### 2.3 Phase 2: Prototype Development

A design brief was created, integrating insights from Phase 1, to guide the development process and ensure team alignment (Supporting Information 2) [29]. Regular meetings among the researchers (SA, YP and ASN) were conducted to discuss the design brief.

High‐fidelity educational resource prototypes, defined as prototypes closely resembling the final product in both appearance and functionality [30], were developed by the researcher (SA) using Canva (https://www.canva.com) and Microsoft Word (version 2408, 2025) between December 2024 and April 2025. Content was informed by current literature, MS organisation websites, and the *Handbook of Clinical Nutrition and Dietetics* (7th ed.) [31], with input from two Accredited Practising Dietitian (APD) researchers working in MS. All materials followed Universal Design for Learning (UDL) principles to support diverse learning needs [32]. The PEMs were designed in accordance with the Centers for Disease Control and Prevention (CDC) disability guidance [33]. The PEMs followed benchmarks for readability (grade level ≤ 8), understandability and actionability (score > 70%), and clarity (score ≥ 90%) using the Hemingway Editor [34], the Patient Education Material Assessment Tool for printable materials (PEMAT‐P) [35], and the CDC Clear Communication Index Score Sheet (CDC CCISS) [36], respectively.

In April 2025, the researcher (SA) built a prototype website using a free website builder (https://www.wix.com) and uploaded the materials. The website's design and functionality adhered to the Web Content Accessibility Guidelines (WCAG) 2.1 level AA guidelines [37]. A senior researcher (YP) and a dietitian‐science communicator (SM) reviewed the material. Feedback on spelling, grammar, and content was incorporated accordingly.

### 2.4 Phase 3: Usability Testing and Prototype Refinement

#### 2.4.1 Participant Sampling and Recruitment

In May 2025, the Phase 1 key stakeholders were invited by email to provide feedback on the educational resource prototypes. Eligible Phase 1 participants who were unable to attend (plwMS, *n* = 8; HCPs, *n* = 5) and those who later expressed interest (plwMS, *n* = 2) were also invited. Additional HCPs were invited, as described in Phase 1, to ensure that the resources were relevant to a diverse range of HCPs. Following Lambert et al.‘s recommendations [38], we continued to recruit plwMS to obtain diverse feedback (e.g., gender, disease stage, education level). All participants completed the same pre‐interview questionnaire used in Phase 1.

#### 2.4.2 Data Collection

Usability testing was conducted through semi‐structured interviews with key stakeholders. The website was designed for use by HCPs in practice, while the PEMs were intended to support patient education. Accordingly, HCPs tested the website and HCP‐directed materials, while plwMS tested the PEMs. The resource prototypes tested comprised high‐fidelity draft prototypes developed in Phase 2.

Prototypes were shared a few days prior to interviews for optional review, with participants informed that the resources were in draft format, open to feedback and not for redistribution. Despite the potential for rehearsed responses/answers [6], this method aimed to facilitate participants’ reflection on their experiences.

Interviews were facilitated by the researcher (SA), enabling participants to navigate the prototypes and provide feedback. PlwMS could review the PEMs in either a printed or digital format. A semi‐structured interview guide (Supporting Information 3), pilot‐tested for face validity with a senior researcher (ASN), was used with probing questions and reflective prompts. Prototype refinements were made iteratively during Phase 3 based on usability testing feedback, with major suggestions (e.g., colour changes, added content) confirmed in later interviews.

Online interviews were conducted and recorded via Zoom, and face‐to‐face interviews were also recorded using Zoom. Usability testing was conducted over 8 weeks between May and June 2025.

### 2.5 Data Analysis

#### 2.5.1 Phase 1: Exploration of Needs and Challenges With Collaborative Ideation

Workshop sticky notes and interview transcripts were analysed thematically by the research team using Braun and Clarke's six‐step inductive reflexive thematic analysis [39]. The notes were reviewed multiple times for familiarity, then manually grouped and coded by the researcher (SA), with input from the research team. Interview transcripts were similarly coded by (SA) using concise phrases, which were translated into sticky‐note style units to facilitate integration with workshop data. Two independent researchers (YP and ASN) coded one transcript and discussed their interpretations to support reflexive dialogue rather than to reach consensus [40]. Codes from workshops and interviews were combined and clustered into key themes. These codes and themes were visually mapped, discussed with the research team, and refined through collaborative analysis.

Similar responses from HCPs and plwMS were grouped into personas. A persona is a fictional character that reflects a broader group of users who share common backgrounds, perspectives, needs, or feelings [41]. It is a tool commonly used in design and product development to ensure newly developed products and/or services are user‐centred [42]. The personas, along with key theme–based design considerations, informed prototype development. It is noted that certain persona features may not be generalisable beyond this context.

Following Phase 1, two five‐page booklets (one for HCPs and one for plwMS) summarising key outcomes were emailed to participants to encourage reflection and support iterative, collaborative contributions. These booklets were not prototypes, but rather synthesis documents of Phase 1 findings. Feedback received from five plwMS and six HCPs was incorporated, alongside Phase 1 findings, to inform the development of draft prototypes.

#### 2.5.2 Phase 3: Usability Testing and Prototype Refinement

Firstly, participants’ feedback was summarised and discussed in frequent meetings with the senior researcher (YP). Phase 3 interviews were analysed by (SA) using the same inductive coding approach. ASN and YP independently coded two transcripts to support analytic dialogue and team engagement [40]. Similar to Phase 1, codes from HCPs and plwMS were combined based on similarities. Themes were generated inductively, visually mapped with related codes, and then discussed and refined with the research team. Insights from Phase 3 guided iterative refinement of the prototypes, which informed the final versions of the educational resources.

#### 2.5.3 Reflexivity

To ensure trustworthiness and rigour, the research team engaged in reflexive discussions throughout all phases of analysis [43]. Visual thematic maps documented analytic decisions, and collaborative team discussions informed iterative refinements, which together formed the audit trail for the analysis. Regular team meetings, a consistent inductive coding approach, and the use of visual maps helped maintain analytic consistency across phases and facilitated the integration of workshop and interview data.

### 2.6 Ethical Considerations

This study was approved by the University of Wollongong Human Research Ethics Committee (2023/307). Informed consent was obtained from all participants before the workshops and interviews commenced. PlwMS were remunerated with a AU$60 gift voucher for their involvement in the workshop (Phase 1) and an AU$30 gift voucher for their involvement in the interviews (Phase 3). Before data analysis, the researcher (SA) checked the accuracy of the sticky notes and transcripts against the audio recordings for quality assurance [44]. The data were de‐identified by removing identifiable information, with any such details in quotes replaced by {X} to ensure anonymity. All quotes were labelled according to a participant number, stakeholder group and years living with MS (e.g., P01, PlwMS, 1 year) or years of experience in MS care (e.g., P01, Dietitian, 1 year).

## 3 Results

### 3.1 Phase 1: Exploration of Needs and Challenges With Collaborative Ideation

#### 3.1.1 Participant Characteristics

A total of 19 HCPs participated, nine attended a 2‐h workshop, and 10 completed individual interviews (average duration: 29.8 ± 14.03 min/interview). Additionally, 14 plwMS participated in a separate 2‐h workshop. Two HCPs and three plwMS provided consent but were unable to attend. No family members or carers joined the workshop. See Table 1 for participants’ self‐reported characteristics.

**Table 1:** Characteristics of the participants in Phase 1.

	HCP (*n* = 19)	PlwMS (*n* = 14)
Sex, *n* (%)		
Female	17 (89.5%)	11 (78.6%)
Male	2 (10.5%)	3 (21.4%)
Gender, *n* (%)		
Woman	16 (84.2%)	11 (78.6%)
Man	2 (10.5%)	3 (21.4%)
Non‐binary/Third gender	1 (5.3%)	0
Age group (years), *n* (%)		
20–24	0	0
25–34	5 (26.3%)	0
35–44	8 (42.1%)	5 (35.7%)
45–54	4 (21.0%)	4 (28.6%)
55–65 +	2 (10.5%)	5 (35.7%)
Highest degree or level of education, *n* (%)		
Certificate III‐IV	0	2 (14.3%)
Diploma	1 (5.3%)	1 (7.1%)
Bachelor's degree (including Hons)	5 (26.3%)	8 (57.1%)
Graduate Diploma	1 (5.3%)	0
Postgraduate degree (MSc and PhD)	11 (57.9%)	3 (21.4%)
Fellowship programme	1 (5.3%)	—
Location, *n* (%)		
New South Wales	9 (47.4%)	4 (28.6%)
Victoria	5 (26.3%)	6 (42.9%)
Queensland	2 (10.5%)	3 (21.4%)
Western Australia	2 (10.5%)	0
South Australia	1 (5.3%)	1 (7.1%)
Practice setting^a^, *n* (%)		
Academic institution	3 (15.8%)	—
Individual practice/sole practitioner	1 (5.3%)	—
MS organisations	3 (15.8%)	—
Primary or community‐based care	3 (15.8%)	—
Private practice	9 (47.4%)	—
Public inpatient setting	1 (5.3%)	—
Public outpatient setting	6 (31.6%)	—
Professionals experience working in MS settings (years)		
Median	7	—
Range	0.5−40	—
Employment status, *n* (%)		
Employed	—	9 (64.3%)
Disability pension	—	3 (21.4%)
Transit disability	—	1 (7.1%)
Retired	—	1 (7.1%)
Type of MS, *n* (%)		
Relapsing‐remitting MS	—	11 (78.6%)
Primary‐progressive MS	—	1 (7.1%)
Secondary‐progressive MS	—	2 (14.2%)
Time since diagnosis (years)		
Median	—	7
Range	—	0.5‐34
Currently on DMTs, *n* (%)		
Yes	—	11 (78.6%)
No	—	3 (21.4%)
PDDS level		
Median	—	3
Range	—	0‐7

Abbreviations: DMTs, Disease Modifying Therapies; HCPs, healthcare professionals; Hons, honours; MS, multiple sclerosis; MSc, Master of Science; PDDS, Patient Determined Disease Steps; plwMS, people living with MS; PhD, Doctor of Philosophy.

^a^ Indicates multiple responses were permitted. —, not applicable.

#### 3.1.2 Personas and Key Themes

Thematic analysis generated four key themes: (1) Addressing healthcare barriers to providing nutrition education in MS care; (2) Supporting person‐centred nutrition communication; (3) Fostering equity through accessible and inclusive MS‐specific educational resources; and (4) The integral role of the dietitian in MS care. These themes, along with 20 key considerations (Table 2), reflect the perspectives of both HCPs and plwMS. Supporting quotes are provided in Supporting Information 4: Tables 1–4.

**Table 2:** Key themes and the 20 key considerations for supporting nutrition in routine MS care (Phase 1).

Themes	Key considerations
**Addressing healthcare barriers to providing nutrition education in MS care**	Addressing educational gaps, including MS‐specific PEMs, misinformation and understanding among plwMS, non‐dietitian HCP knowledge and interest, as well as dietitian training in MS. Overcoming healthcare systematic barriers, including consultation time constraints, limited access to dietitians, and the cost of dietetic services. Bridging knowledge evidence gaps, including the need for research and guidelines on MS and diet.
**Supporting person‐centred nutrition communication**	Considering plwMS’ readiness and motivation to receive nutrition advice. Respecting plwMS’ choices and preferences. Providing practical and realistic dietary options.
**Fostering equity through accessible and inclusive MS‐specific educational resources**	Including specific nutrition and diet topics. Designing credible educational resources. Ensuring educational resources are easily accessible. Providing blended learning resources for HCPs. Designing digital, printable PEMs. Creating simple, easy‐to‐understand PEMs. Including visual aids in PEMs. Designing PEMs with clear visual formatting. Offering audio‐format PEMs. Representing diverse backgrounds and cultures in PEMs.
**The integral role of the dietitian in MS care**	Access to individualised nutrition advice. Supporting sustained dietary behaviour change. Promoting interdisciplinary collaboration through dietitian referrals. Raising awareness about the role of dietitians in MS care.

Abbreviations: HCPs, healthcare professionals; MS, multiple sclerosis; PEMs, patient education materials; plwMS, person living with MS.

**Figure 3:**
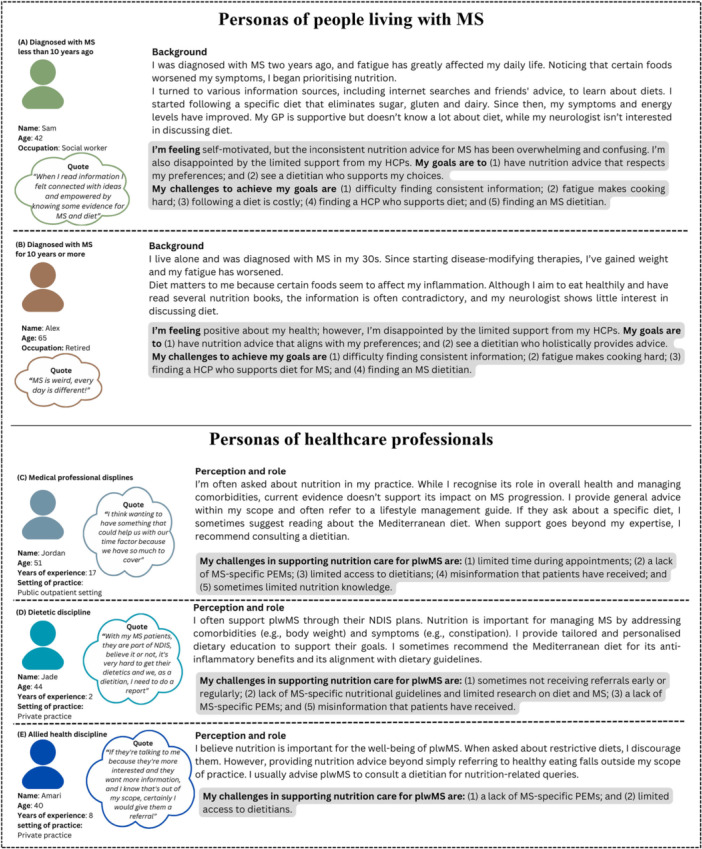
Personas generated during Phase 1. GP, practitioner; HCPs, healthcare professionals; MS, multiple sclerosis; NDIS, National Disability Insurance Scheme; PEMs, patient education materials; plwMS, people living with MS.

Two gender neutral plwMS personas emerged, reflecting varied dietary experiences, which were strongly influenced by their stage of disease (Figure 3):

- **Persona A–Sam (diagnosed** < **10 years):** Follows a specific diet that eliminates specific foods and seeks nutrition advice that respects their preferences.
- **Persona B–Alex** (**diagnosed** ≥ **10 years):** Aims to eat a generally healthy diet and values holistic nutrition advice (e.g., MS and overweight/obesity).

Despite differences, both groups shared common challenges, related to navigating nutrition information (Theme 1), accessing person‐centred guidance (Theme 2), the need for accessible and tailored resources (Theme 3), and obtaining personalised nutrition support from dietitians (Theme 4).

Three HCP gender neutral personas reflected discipline‐specific views (Figure 3):

- **Persona C–Jordan** (medical discipline, including physicians and nurses): Recognises the role of nutrition in general health and comorbidity management but perceives limited evidence for its impact on MS progression. They consider providing general advice within their scope‐of‐practice.
- **Persona D–Jade** (dietetic discipline): Views nutrition as essential for managing MS‐related complications and provides personalised advice. Sometimes they recommend the Mediterranean diet for its consistency with dietary guidelines.
- **Persona E–Amari** (allied health discipline, excluding dietitians): Values nutrition for overall well‐being but advises plwMS to consult dietitians, as providing specific advice falls outside their scope‐of‐practice.

These personas reflect profession‐specific barriers (Theme 1) and the approaches HCPs take when communicating about nutrition (Theme 2).

##### 3.1.2.1 Theme One: Addressing Healthcare Barriers to Providing Nutrition Education in MS Care

PlwMS described using nutrition as a self‐management strategy, though many felt unsupported by HCPs. For example, Sam (Persona A), felt overwhelmed by conflicting nutrition information and unsupported in following a specific diet. HCPs across disciplines, including dietetics, reported barriers to providing nutrition information, including limited MS‐specific PEMs (Persona C, Persona D and Persona E). Misinformation and pre‐existing dietary beliefs among plwMS were also seen by HCPs as barriers to delivering evidence‐based nutrition advice (Jordan–Persona C) and (Jade–Persona D). As a result, key stakeholders perceived a need for MS‐specific PEMs to overcome these educational gaps.

Some HCPs acknowledged limitations to their own nutrition knowledge, such as Jordan (Persona C) and Jade (Persona D), to advise plwMS on nutrition. Other HCPs noted a lack of interest in nutrition among HCP colleagues (e.g., physicians). Furthermore, plwMS perceived that dietitians often lacked MS‐specific nutrition knowledge, leading HCPs to recommend targeted professional training. Most HCPs across disciplines expressed a desire for further learning to better support the nutritional needs of plwMS. Additionally, many dietitians (Jade–Persona D) and plwMS voiced frustration over the limited evidence base for MS‐specific nutrition, advocating for more research and development of practice guidelines.

Consultation time constraints, especially among medical professionals (Jordan–Persona C), were noted as a challenge to providing nutrition advice. PEMs were suggested as a tool to support information delivery outside consultations. Across groups, including plwMS, medical (Jordan–Persona C), and allied health (Amari–Persona E) professionals, described challenges in accessing dietitians. Suggested solutions included increased government funding (e.g., National Disability Insurance Scheme (NDIS)), telehealth and free initial consultations.

##### 3.1.2.2 Theme Two: Supporting Person‐Centred Nutrition Communication

HCPs acknowledged the importance of considering patient readiness and motivation when providing nutrition advice, though their approaches varied. While a few HCPs, such as Jordan (Persona C), proactively initiated nutrition conversations, most reported waiting for patients to raise the topic. Advice ranged from general lifestyle tips (Jordan–Persona C) to recommendations aligned with dietary guidelines or the Mediterranean diet (Jade–Persona D).

PlwMS diagnosed within the last 10years (e.g., Sam–Persona A) appeared more self‐motivated than those with longer‐standing MS (e.g., Alex–Persona B) and prioritised dietary management. However, both groups reported limited support from their HCPs and emphasised the need for personalised guidance that respects individual preferences and circumstances. Respecting dietary choices was seen as essential for plwMS’ dignity and autonomy. For example, HCPs highlighted the importance of empowering plwMS with credible information and realistic support, even when providing nutrition advice falls outside their scope (e.g., Amari–Persona E). They acknowledged their responsibility in informing plwMS about the potential risks of restrictive diets and the benefits of sustainable dietary changes.

Person‐centred language in PEMs was recommended, including listing food options and alternatives rather than prescriptive advice. HCPs also suggested framing nutrition messages to reflect evidence‐based benefits without overpromising.

##### 3.1.2.3 Theme Three: Fostering Equity Through Accessible and Inclusive MS‐Specific Educational Resources

Key stakeholders identified the need for credible, accessible nutrition educational resources for both plwMS and HCPs. They emphasised referencing scientific evidence and involving qualified professionals (e.g., dietitians) to build trust. Many suggested hosting resources on a website with separate sections for HCPs and plwMS, ideally accessible via reputable platforms (e.g., MS Australia).

HCPs proposed several methods to improve access to MS‐related nutrition information, such as webinars with continuing professional development hours, a summary booklet/sheet, and conference workshops to support HCP training. Key stakeholders also recommended offering PEMs in printable and downloadable formats (e.g., fact sheets, pamphlets), with quick response (QR) codes or universal resource locators (URLs) for easy sharing. While most key stakeholders preferred PEMs in printable format, some HCPs noted that younger adults might prefer short videos or episodes.

Clear, inclusive design of PEMs was seen as essential. Key stakeholders stressed using plain language to support varying health literacy levels and incorporating accessibility features (e.g., bright colours, large fonts, audio formats). Visual aids like infographics and culturally diverse food examples were recommended to enhance understanding and acknowledge the varied backgrounds of plwMS.

##### 3.1.2.4 Theme Four: The Integral Role of the Dietitian in MS Care

Despite challenges in accessing dietetic services and perceived gaps in dietitians’ MS‐specific nutrition knowledge, key stakeholders valued dietitians as essential members of the MS healthcare team. HCPs emphasised the need for individualised nutrition plans to manage comorbidities, MS symptoms, and risks linked to restrictive diets, reinforcing that generic nutrition advice is insufficient. PlwMS appeared to value dietitians with MS‐specific expertise (e.g., Sam–Persona A) and those who could provide holistic advice (e.g., Alex–Persona B). Barriers such as fatigue and food costs influenced the feasibility of long‐term dietary changes among plwMS, reinforcing the need for personalised counselling. While some HCPs suggested including actionable tips to modify behaviour in PEMs, they emphasised the importance of ongoing dietitian support for sustained change.

Most HCPs emphasised the need for interdisciplinary collaboration, with referrals to dietitians by non‐dietitian HCPs (e.g., Jordan–Persona C; Amari–Persona E) reflecting an awareness of their own scope‐of‐practice. As a result, HCPs recommended including referral guidance in the resource, such as screening tools, checklists and information on NDIS and Medicare eligibility. Finally, raising awareness of dietitians’ roles in MS care, especially among neurologists and general practitioners (GPs), was seen as vital for improving referrals and coordinated care.

### 3.2 Phase 2: Prototype Development

**Figure 4:**
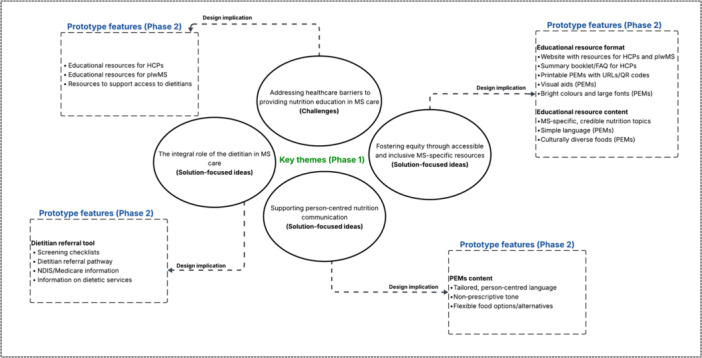
Conceptual diagram illustrating the connection between Phase 1 thematic insights and Phase 2 prototype design features. FAQs, Frequently Asked Questions; HCPs, healthcare professionals; MS, multiple sclerosis; NDIS, National Disability Insurance Scheme; PEMs, patient education materials; plwMS, people living with MS; QR, code generator; URLs, Uniform Resource Locators.

The MS‐specific nutrition educational resource prototypes were fully developed into tangible resources, informed by Phase 1 theme–based design considerations, including content and format (Figure 4). The prototypes included a set of PEMs, a self‐guided summary document for HCPs featuring a question‐and‐answer (Q&A) format, and a dietitian referral screening checklist and pathway. These nutrition resources were designed to meet the needs identified in Phase 1 among both plwMS and HCPs across various disciplines and were hosted on a dedicated website.

**Figure 5:**
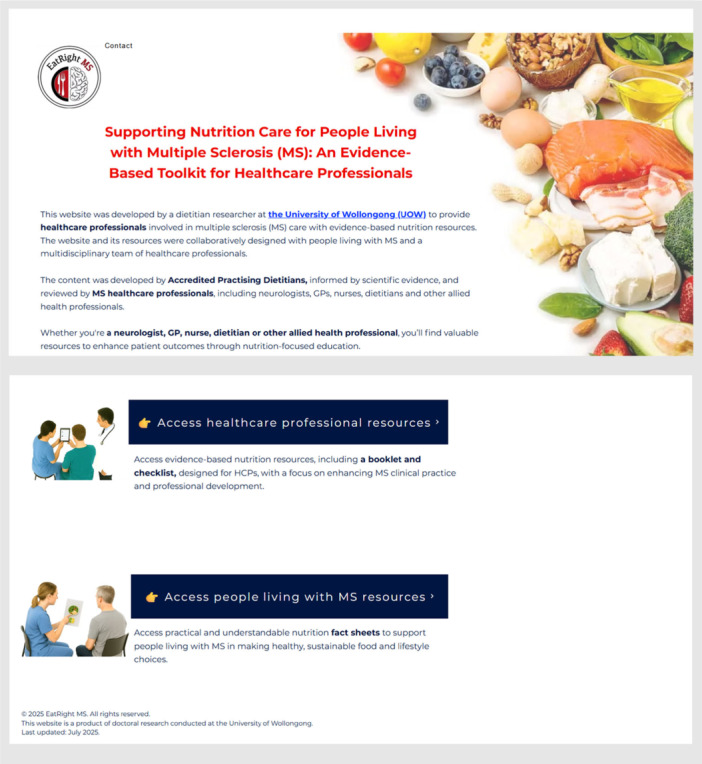
Screenshot of the website homepage displaying two main sections.

The website consists of two main sections: (1) a PEMs section with MS‐specific nutrition fact sheets to guide plwMS in routine care, and (2) a learning section for HCPs to empower them to confidently address patient questions and promote interdisciplinary collaboration (Figure 5). All materials include downloadable links (URLs).

While the content is grounded in established dietary recommendations, specifically the Australian Dietary Guidelines [45], it was tailored to incorporate emerging MS‐specific nutrition topics supported by scientific evidence. The PEMs highlighted the benefits of dietary choices, focusing on positive outcomes rather than the consequences of non‐adherence. This approach ensures the use of MS‐relevant, evidence‐based language, while providing practical, actionable guidance for both HCPs and plwMS.

Resource credibility was established by clearly stating that APDs were involved in the creation and review of materials, and by indicating the date of the most recent update [38]. Additional credibility was provided by embedding links and QR codes directing users to further scientific information, such as MS Australia's 2025 Lifestyle Management Guides [46]. A copyright notice was added to establish ownership.

### 3.3 Phase 3: Usability Testing and Prototype Refinement

#### 3.3.1 Participant Characteristics

A total of 18 HCPs and 15 plwMS participated in the usability testing. Of these, ten HCPs and eight plwMS were involved in the Phase 1. Four plwMS reported having varying degrees of neurodiversity. See Table 3 for participant self‐reported characteristics.

**Table 3:** Characteristics of the participants in Phase 3.

	HCP (*n* = 18)	PlwMS (*n* = 15)
Sex, *n* (%)		
Female	13 (72.2%)	13 (86.7%)
Male	5 (27.8%)	2 (13.3%)
Gender, *n* (%)		
Woman	12 (66.7%)	13 (86.7%)
Man	5 (27.8%)	2 (13.3%)
Non‐binary/Third gender	1 (5.6%)	0
Age group (years), *n* (%)		
20–24	0	0
25–34	6 (33.3%)	1 (6.7%)
35–44	4 (22.2%)	4 (26.7%)
45–54	6 (33.3%)	5 (33.3%)
55–65 +	2 (11.1%)	5 (33.3%)
Highest degree or level of education, *n* (%)		
Certificate III‐IV	0	1 (6.7%)
Diploma	0	3 (20.0%)
Bachelor's degree (including Hons)	8 (44.4%)	4 (26.7%)
Graduate Diploma	1 (5.6%)	4 (26.7%)
Postgraduate degree (MSc and PhD)	6 (33.3%)	3 (20.0%)
Fellowship programme	3 (16.7%)	—
Location, *n* (%)		
New South Wales	10 (55.6%)	3 (20.0%)
Victoria	4 (22.2%)	4 (26.7%)
Queensland	2 (11.1%)	4 (26.7%)
Western Australia	1 (5.6%)	2 (13.3%)
South Australia	1 (5.6%)	1 (6.7%)
Tasmania	0	1 (6.7%)
Practice setting^a^, *n* (%)		
Academic institution	2 (11.1%)	—
Individual practice/sole practitioner	1 (5.6%)	—
MS organisations	3 (16.7%)	—
Primary or community‐based care	2 (11.1%)	—
Private practice	7 (38.9%)	—
Public inpatient setting	3 (16.7%)	—
Public outpatient setting	6 (33.3%)	—
Professionals experience working in MS settings (years)		
Median	10.5	—
Range	1−27	—
Employment status, *n* (%)		
Employed	—	10 (66.7%)
Disability pension	—	4 (26.7%)
Transit disability	—	1 (6.7%)
Type of MS, *n* (%)		
Relapsing‐remitting MS	—	12 (80.0%)
Primary‐progressive MS	—	3 (20.0%)
Secondary‐progressive MS	—	0
Time since diagnosis (years)		
Median	—	5
Range	—	0.25‐32.5
Currently on DMTs, *n* (%)		
Yes	—	11 (73.3%)
No	—	4 (26.7%)
PDDS level		
Median	—	2
Range	—	0‐7

Abbreviations: DMTs, Disease Modifying Therapies; HCPs, healthcare professionals; Hons, honours; MS, multiple sclerosis; MSc, Master of Science; PDDS, Patient Determined Disease Steps; plwMS, people living with MS; PhD, Doctor of Philosophy.

^a^ Indicates multiple responses were permitted. _—_, not applicable.

Interview durations averaged 33.2 ± 12.96 min for HCPs and 47 ± 12.8 min for plwMS. Three interviews were conducted face‐to‐face (2 HCPs, 1 plwMS), while the remainder were held online. Prior to the interviews, 10 HCPs and 11 plwMS navigated all the provided prototypes. During the interviews, all plwMS reviewed the five PEMs, while 13 HCPs reviewed both the PEMs and HCP‐targeted materials, including the website.

#### 3.3.2 Key Themes

Thematic analysis generated four key themes: (1) Clear, targeted messaging as a driver of action; (2) Visually engaging and informative design; (3) Lived experiences informing choices; and (4) Building relationships through trust, credibility and connection. These themes reflect the feedback provided by both HCPs and plwMS. Supporting quotes are provided in Supporting Information 5: Tables 1–4.

##### 3.3.2.1 Theme One: Clear, Targeted Messaging as a Driver of Action

Most plwMS found the PEMs informative, with many appreciating new content, while others felt validated in their existing knowledge. Those with advanced nutrition understanding found some of the content familiar rather than new, while many newly diagnosed plwMS wanted the relevance of general nutrition sections (e.g., food groups) to be stated more explicitly in relation to MS. Preferences for content length varied among plwMS, with suggestions to reduce repetition to avoid cognitive overload. Individual learning styles influenced preferences for either concise or detailed content. Suggestions from plwMS included rewording and clarifying specific sentences for improved readability and clarity, defining clinical terms such as ‘metabolic’, and including food label reading.

HCPs described the website and booklet as comprehensive and useful, particularly for enhancing their confidence in guiding plwMS. They perceived PEMs as valuable for patient education, especially during time‐limited consultations. HCPs suggested improvements such as rewording, reorganising content and enhancing clarity. The dietitian referral screening tool was considered helpful, especially for nurses and GPs, with recommendations to make it more person‐centred and include relevant questions (e.g., mental health and physical/cognitive barriers), and expanded referral guidance (e.g., funding options).

Both groups valued practical information. For example, plwMS valued the QR code, links and shopping lists that supported application in daily life. Similarly, HCPs found links to access relevant information (e.g., dietetics services) and practical tips around providing nutrition advice relevant to their routine practice.

##### 3.3.2.2 Theme Two: Visually Engaging and Informative Design

Visual elements of the PEMs, such as tables, headings, bullet points, and bold text, were well received by key stakeholders for helping communicate key messages. The inclusion of signposting and summaries in the PEMs was also valued for improving content organisation. PlwMS found visuals engaging and helpful for understanding, though some suggested improving sections lacking headings or images. PlwMS who noticed brand names in the shopping list appreciated them, as it was perceived to increase their confidence when shopping compared to generic food products.

HCPs valued professional design, recommending minimal use of images and colours in the HCP booklet. Suggested improvements included clearer formatting, larger QR codes, and better alignment of visual elements. The website was described as visually appealing and easy to navigate, with distinct sections. Recommendations included automatically linking each section in the booklet's Table of Contents to its corresponding page and listing PEMs on the website in a single row to reduce scrolling.

A majority of plwMS preferred the PEMs in a printable format. In line with this, both HCPs and plwMS suggested removing the dark background to make the PEMs more accessible and print friendly. While plwMS preferred separate fact sheets to avoid information overload, some HCPs recommended combining them into a single booklet, with some suggesting printing them in advance for use in clinical settings.

##### 3.3.2.3 Theme Three: Lived Experiences Informing Choices

Key stakeholders valued the PEMs for supporting personalised decision‐making, praising their flexibility in dietary options (e.g., swaps and recipes). Some key stakeholders described the tone of the PEMs as non‐judgmental, promoting autonomy and enjoyment of food. Suggestions included adding more options, such as lactose‐ and gluten‐free choices, culturally diverse meals and dining‐out tips, to reinforce flexibility in the PEMs. Furthermore, some plwMS recommended including a note to emphasise personalisation based on individuals’ preferences and needs.

HCPs appreciated the website's flexible, self‐directed format, allowing them to select resources needed for their workflow. This autonomy was seen as beneficial for professional learning and encouraged use in routine practice.

##### 3.3.2.4 Theme Four: Building Relationships Through Trust, Credibility and Connection

Credibility and trustworthiness appeared to influence key stakeholder confidence in the resources. Key stakeholders valued the involvement of dietitians (APDs) and the use of up‐to‐date information, which contributed to a sense of trust in using and/or sharing the resources. To further strengthen trust, a few plwMS suggested including a statement about MS community involvement in the PEM design, while some HCPs recommended referencing contributions from HCPs like neurologists and naming the organisation behind the website.

Key stakeholders also emphasised the value of referencing other HCPs within the resources. While practical nutrition tips were appreciated, they suggested including referral information for psychologists, exercise physiologists and dietitians in PEMs. Additional recommendations included adding guidance on accessing relevant services through NDIS or MS organisations, and reinforcing dietetic access pathways.

#### 3.3.3 Prototype Refinement

To enhance the educational resource prototypes, modifications were made according to insights generated from Phase 3.

## 4 Discussion

This study described a design process for new nutrition educational resources in MS care. This collaborative approach, engaging key stakeholders, has led to the co‐development of user‐centred, practical solutions for integrating nutrition into routine MS care. Future adoption of these co‐developed nutrition educational resources into practice could facilitate integration of nutrition‐related care into MS care. Thus, interpretation of usability testing findings was informed by Rogers’ Diffusion of Innovation theory [47], which identifies five factors influencing adoption: relative advantage, compatibility, complexity, trialability and observability. Addressing current educational barriers and refining the resources to meet end‐user needs required an understanding of such adoption factors.

Educational barriers, specifically gaps in PEMs and HCP learning/training, remain challenges to supporting nutrition‐related care in MS care [4, 48]. To address this, a digital platform (website) was suggested as a preferred modality for MS‐specific nutrition resources that HCPs can use in practice. This preference is likely driven by the demand for accessible and flexible resources [4]. The website can be defined as a toolkit [49] aiming to improve the consistency of evidence‐based nutrition advice provided to plwMS, as highlighted in the HCP personas in this study. While toolkits are generally useful to deliver health information, they often do not specify their evidence base [49]. Thus, the co‐developed toolkit presented as an output of this study explicitly states that it is evidence‐based, and includes references to ensure credibility, which was highly valued by the key stakeholders during usability testing. This likely reflects a perception of relative advantage [47], as potential users perceived clear benefits in using MS‐specific, evidence‐based nutrition educational resources compared to previously available options, such as generic or non‐specific PEMs.

In our study, plwMS emphasised the importance of being involved in their own nutrition care. Communication elements they valued, such as respecting preferences and offering options, broadly align with principles of shared decision‐making, which can promote respect and autonomy, and may increases trust [50] and improve health outcomes [51]. Given this, future HCP training efforts should focus on strategies that facilitate shared decision‐making, allowing evidence‐based nutrition guidance to align with individual goals. For instance, discussing the benefits and risks of specific diets can empower plwMS without overriding their choices. The co‐developed HCP booklet emphasised this approach, reinforcing that nutrition advice in MS care must be individualised rather than a one‐size‐fits‐all.

Interestingly, our study found that plwMS needed nutrition information from their HCPs, while also actively participating in dietary decisions. As both newly diagnosed and those with long‐lasting MS preferred adaptable guidance, person‐centred nutrition PEMs were designed to be broadly applicable. Usability testing confirmed that flexible, reliable resources supported nutrition‐informed choices tailored to personal circumstances. However, newly diagnosed individuals expressed a need for clearer links between general advice and MS relevance. This finding guided iterative refinement of the PEM content to increase relatability. This aligns with the compatibility and relative advantage [47], as users perceived the need for nutrition resources to be respectful of their needs and values, ultimately boosting the perceived advantage over generic resources. Similarly, the website's flexibility is likely to enhance compatibility with HCPs’ different clinical roles and workflows. These perceptions suggest potential for educational resource adoption [47].

Usability testing revealed that including branded food product images in PEMs can improve understanding and confidence in making informed choices. Similar findings have been reported in previous research exploring nutrition‐related PEMs [38], where the authors advocate for the use of images with brand names to support understanding. To the authors’ knowledge, no existing legislation in NSW explicitly restricts the inclusion of branded food product images in PEMs.

The co‐developed PEMs aim to meet the nutrition information needs of plwMS while addressing several barriers identified earlier in our study, including limited consultation time (Jordan–Persona C) and challenges in accessing dietetic services. Whilst the PEMs are primarily targeted at plwMS, they can also serve as valuable resources for HCPs during healthcare consultations to improve communication [52]. Usability testing indicated that physicians and nurses perceived the PEMs as time‐saving resources, reflecting the perceived relative advantage of the PEMs [47]. Generally, users are more likely to perceive a tool as timesaving if it is simple. In line with this, usability testing highlighted that high complexity within resources has the potential to hinder educational resource adoption [47]. For example, while the website was considered easy to use by HCPs, navigating the PEMs section was perceived as more complex, potentially limiting access during consultations. Similarly, information overload within the PEMs could reduce ease of use among plwMS. These findings informed iterative refinements focused on simplifying navigation and reducing content overload.

Surprisingly, most plwMS preferred printable PEMs, a preference reinforced during usability testing. This finding is unexpected, given the increasing accessibility of health information online. However, this may be because printed educational resources do not require digital literacy and can help plwMS feel more engaged and responsible for their own care. Additionally, the digital format may impose a higher cognitive burden due to the different ways in which individuals engage with information online [53]. In light of this finding, HCPs should consider these preferences and provide printed PEMs for plwMS, along with online links to prevent loss of access to the materials where possible. Printed materials should also be accessible [52] in terms of colour choices to support readability for all users, as highlighted during the usability testing.

An additional insight during the design process was the need for dietitians’ input to meet the specific nutritional needs of plwMS. To support this, screening questions and a referral pathway were co‐developed and hosted on the website to improve coordination of care when indicated [54]. The HCP booklet emphasised the dietitian's role in providing tailored nutrition plans and facilitating behaviour change [55]. Although the co‐developed PEMs and HCP booklet used actionable recommendations, this alone may not be sufficient to support dietary behaviour changes among plwMS [56]. While the HCP booklet provides only basic MS‐related nutrition information, the content was nonetheless valued by both dietitians and non‐dietitian HCPs, suggesting a perceived relative advantage [47].

Accessing dietetic care is often challenged by factors such as high consultation fees and limited availability [5, 57], corroborated by the findings of the present study. Therefore, an important inclusion in the resources was explicit guidance on how and where to access dietetic services, along with other relevant supports, such as MS organisations. Usability testing reinforced this need, with HCPs emphasising the value of providing clear access information across multiple resources and referring plwMS to community health services or non‐profit MS organisations for subsidised or cost‐free support. Inclusion of access guidance and links to relevant services in subsequent updates of the resources may have strengthened the perceived relative advantage and compatibility, though this was not formally tested in the present study [47].

Based on the Diffusion of Innovation theory [47], an educational resource is more likely to be adopted by key stakeholders when it demonstrates relative advantage and aligns with existing practices and preferences (compatibility). In contrast, if an educational resource presents with complexity, adoption may be hindered [47]. Notably, many key stakeholders navigated the resources independently during their free time prior to interviews, indicating evidence of trialability, which may infer increased likelihood of educational resource adoption [47].

### 4.1 Strengths and Limitations

A key strength of our study was its user‐centred design, involving key stakeholders early and throughout each phase of the design process. Reflexivity within this study was also an important strength. However, our study has its limitations. In the plwMS workshop, only the summary session was audio‐recorded. Although researchers took several steps to preserve context, including capturing key details through structured sticky notes, conducting a post‐workshop debrief with documented observations, engaging participants in reflection and validation through a summary booklet, and collaboratively analysing the workshop data, some nuances of the discussions may not have been fully captured.

Although the study excluded plwMS with severe cognitive impairment, this criterion did not exclude all cognitive challenges. We acknowledge that plwMS may experience varying levels of cognitive challenges that could impact information processing [58] and, consequently, comprehension of PEMs and usability equity. However, we did not collect data on participants’ cognitive impairment levels.

All plwMS who took part in our study had completed education beyond high school. This may have influenced the perceived usability of the PEMs, as individuals with higher health literacy may feel more confident navigating such materials. The participant self‐selection [59], including recruitment of individuals who had engaged in our previous studies, may also have shaped the views shared, as the majority were more motivated and had an interest in nutrition for MS. As a result, the perceived usability and acceptability of the educational resources in this sample may be higher than in harder‐to‐reach groups, including those with lower education, lower health literacy, or less interest in nutrition, which may limit the transferability of findings to the broader MS population. Future research is needed to explore how best to engage harder‐to‐reach groups, including those with severe cognitive impairment, lower health literacy, limited education, or less interest in nutrition.

Additionally, it was not possible to implement or prototype all the ideas generated by key stakeholders due to resource limitations, though this presents opportunities for future research.

### 4.2 Future Directions

Firstly, collaboration with MS organisations is recommended to ensure educational resources are accessible, including links to the co‐developed website on their platforms and dissemination of materials within MS clinics. Secondly, as Phase 3 focused on initial testing to iteratively refine the resources, future research should test the applicability and efficacy of the resources in real‐world practice settings. This would further support the trialability of educational resources [47]. Finally, given that MS‐related nutrition research evidence is still evolving, these resources will need to be reviewed regularly to maintain their currency and relevance over time.

## 5 Conclusion

Our study involved key stakeholders to co‐develop and refine a set of user‐centred, MS‐specific nutrition educational resources (a toolkit) for both HCPs and plwMS hosted on a website to support the integration of nutrition into MS care. These resources have the potential to address commonly identified barriers, including limited consultation time with physicians and nurses, as well as restricted access to dietetic services. Additionally, they may empower plwMS to make informed nutrition choices, reduce confusion caused by misinformation and support their MS management. The resources were generally valued by key stakeholders and appeared to be informative, acceptable and usable. Future research should focus on testing the applicability and efficacy of the resources in real‐world practice settings and evaluate the pathway to adoption.

## Author Contributions

**Shoroog Allogmanny:** conceptualisation, methodology, writing – original draft, writing – review and editing, data curation, formal analysis, investigation, visualisation, software, resources, project administration. **Yasmine Probst:** conceptualisation, methodology, supervision, writing – review and editing, validation, visualisation. **Karen Zoszak:** formal analysis, writing – review and editing, data curation. **Anita Stefoska‐Needham:** conceptualisation, methodology, data curation, supervision, formal analysis, writing – review and editing, validation, visualisation.

## Funding

The authors received no specific funding for this work.

## Ethics Statement

This study was approved by the University of Wollongong Human Research Ethics Committee, New South Wales, Australia (2023/307). Witten and verbal consent informed consent was obtained from all participants.

## Conflicts of Interest

Yasmine Probst is a person living with MS and was funded by Multiple Sclerosis Australia for a research fellowship and has had her research funded by Multiple Sclerosis Australia. Karen Zoszak was receiving a post graduate scholarship from Multiple Sclerosis Australia. Shoroog Allogmanny and Anita Stefoska‐Needham have no conflicts of interest to disclose.

## Supporting information

Supplementary File_Co‐developing educational resources to integrate nutrition into MS care.

## Data Availability

The data that support the findings of this study are available from the corresponding author upon reasonable request. The final resources can be accessed from the corresponding author upon reasonable request.
