# Supplementary material for: Co‐Developing User‐Centred Nutrition Educational Resources to Integrate Nutrition Into Multiple Sclerosis Care: A Collaborative Approach With Healthcare Professionals and Healthcare Consumers
Source: Health Expect. 2026 Apr 2;29(2):e70656. doi: 10.1111/hex.70656 (PMC13051993; doi:10.1111/hex.70656)
Supplement: Supplementary file 1 — Supplementary File_Co‐developing educational resources to integrate nutrition into MS care. [file HEX-29-e70656-s001.docx]

Supporting Information 1: Context for the four headings: (1) background story; (2) satisfaction points; (3) pain points/challenges; and (4) solutions and ideas

**Participant narratives or journeys**

| Background | Identifying the story behind the user’s experience; **plwMS** (e.g., age, years living with MS and then their journey with nutrition); **HCPs** (e.g., profession, practice setting, years practising and their role in supporting nutrition for plwMS). |
| --- | --- |
| Satisfaction points | Discussing what worked well (positive experiences in their journey as a **patient** or in their role as a **HCP** in relation to nutrition and MS). |
| Pain points, challenges | Identifying the pain points or key challenges the user faces; **plwMS (**e.g., when seeking or wanting nutrition guidance from HCPs); **HCPs** (e.g., when delivering or wanting to provide nutrition advice to plwMS). |
| Solutions and ideas | Once pain points or challenges are identified, use the **"How Might We" questions** bridge to start the ideation process and generate solutions. |

**Abbreviations:** MS: multiple sclerosis; plwMS: people living with MS; HCPs: healthcare professionals.

Supporting Information 2: Design brief for MS-specific nutrition educational resources

| **Project name** |
| --- |
| MS-specific nutrition educational resources |
| **Project overview** |
| - Creating a website for HCPs to access MS-specific nutrition educational resources, including PEMs. |
| **Target audience** |
| - HCPs involved in MS care (neurologists, GPs, MS nurses and all allied health professionals, including dietitians). - PlwMS. |
| **Project objectives** |
| - **To support the integration of nutrition into routine MS care.** - **Specifically, this project aims to:** - Enhance HCPs’ MS-related nutrition knowledge through evidence-based learning materials. - Enhance the accessibility and consistency of nutrition information for plwMS through evidence-based nutrition-related PEMs. - Foster interdisciplinary collaboration among HCPs by facilitating referrals to dietitians through referral guidance and a pathway. |
| **Website structure and format** |
| - Two sections:  1. PEMs; including downloadable PEMs. 2. HCP materials; including a downloadable summary booklet and a dietitian referral decision making tool. |
| **Materials content** |
| - **HCP-focused materials**  1. **Evidence-based MS-specific** **nutritional management FAQs with answers and summary guide for HCPs**  - Evidence-based nutrition recommendations for plwMS, including diet and supplements, along with practical tips and a drug-food interactions checker tool. - Online nutrition information searched by plwMS. - Evidence for diets promoted for MS. - Dietitians in MS care. - Additional resources and tools, including MS dietetic services, find a dietitian, behaviour change strategies, modifiable lifestyle management guidance, NDIS and Medicare. - Latest research article references.  1. **Screening questions for dietitian referral decision-making (Step 1: checklist questions; Step 2: pathway)**  - **Five PEMs**   1. Evidence-based nutrition recommendations for plwMS, including gut health and inflammation.   2. Fatigue-friendly kitchen, fatigue-friendly cooking, fatigue-friendly meals and MS-friendly shopping list.   3. Calcium and vitamin D.   4. MS-related Metabolic health (comorbidities).   5. MS-related symptom management. |
| **Compliance and accessibility requirements** |
| - **Website compliance** - Ensure adherence to the WCAG 2.1 Level AA, ensuring accessibility for all HCP users. - Computer, mobile and tablet-friendly design to ensure accessibility on different devices. - **HCP learning materials compliance** - Follow the UDL guidelines to meet different learning needs. - **PEMs compliance** - Follow the UDL guidelines to meet different learning needs. - Ensure compliance with disability guidance provided by the CDC to enhance inclusivity. - Maintain a reading level of U.S. grade 8 or lower, using the Hemingway Editor. - Maintain an understandability and actionability score of >70%, as measured by the PEMAT-P. - Maintain a clarity score of ≥90%, using the CCISS. |
| **Website functionality requirements** |
| - Include easy and simple navigation. - Enable easy PDF downloads for print-friendly versions. - Provide active URLs linking directly to the same PDFs for easy access and sharing online. - The website prototype should be live with essential features for usability testing. |
| **Visual requirements** |
| - **Font and background colours**: Use EatRight MS logo for both website and materials and ensure there is sufficient colour contrast between background and text. - **Font style**: Use sans serifs fonts for both website and materials (e.g. Calibri, Arial, Helvetica) with left-aligned and 1.5 or double line spacing and at least double spacing after paragraphs. - **Font size:** Use 12-14 to the body text and 16-18 to the headings (materials) and a minimum font size of 16 px for body text and 18–24 px for headings (website). - **Images and visuals**: Ensure images represent diversity, including different people and foods to reflect inclusivity. |
| **Budget** |
| In kind |
| **Timeline** |
| - Material and website prototype development: December 2024 to April 2025 - Usability testing and feedback collection from HCPs and plwMS: May to June 2025. - Iterative refinement based on the feedback: June 2025. - Finalising final versions: June to July 2025. |

**Abbreviations list**

CDC: Centers for Disease Control and Prevention.

CCISS: Clear Communication Index Score Sheet.

FAQs: Frequently Asked Questions

GPs: General Practitioners.

HCPs: Healthcare Professionals.

MS: Multiple Sclerosis.

NDIS: National Disability Insurance Scheme.

PEMAT-P: Patient Education Materials Assessment Tool (Printable Version).

PEMs: Patient Education Materials.

PlwMS: People Living with Multiple Sclerosis.

US: United State.

URLs: Uniform Resource Locators.

WCAG: Web Content Accessibility Guidelines.

Supporting Information 3: Semi-structured question guide used in usability testing interviews

| - Welcoming the participants and introducing the moderator. - Providing a brief overview of the research, including its background and purpose. - Discussing recording and consent, emphasising privacy, confidentiality and data storage procedures. |
| --- |
| Start recording  **Part 1 (Navigation and initial reaction)**  Before we begin, have you had a chance to look through the resources I sent you. It's absolutely fine if you haven't had the time or prefer not to; you can navigate through them now while we talk.   - What is/was your general impression of the resource? |
| **Part 2 (Feedback on PEMs (plwMS), HCP materials and website (HCPs))**   - What do you like about the content? - What don’t you like about the content? How would you change or improve….*(mention identified aspect/s)?* - What information did you find most valuable/important/helpful? - Was there any information that was of no interest to you? - What do you like about the format/design? - What don’t you like about the format/design? How would you change or improve….*(mention identified aspect/s)?* - Do you have any suggestions to improve the content or design of the resources?   Key aspects for probing questions, only if needed:  Content (relevance, usefulness, language, clarity, understandability, actionability, trustworthiness)  Format (navigation, layout, organisation, visual, colour, accessibility, pictures of food) |
| **Part 3 (A quick reflection)**   - Just before we finish, how did you engage with the resource? For example, did you read it word for word, or did you mostly scan the highlights, look at images, or focus on particular sections? (plwMS) - Just before we finish, how did you use or interact with the website while navigating it? For example, did you explore it in order or focus on certain parts? and how do you see yourself using these resources in your practice? (HCPs) |
| Thank you for participating and sharing your feedback today. Your contributions are incredibly valuable and will help us improve the resources. |

**Abbreviations.** HCPs: healthcare professionals; PEMs: patient education materials; plwMS: people living with multiple sclerosis.

Supporting Information 4: Supporting quotes illustrating the 20 key considerations in support of each key theme (Phase 1)

**Table 1** Participant quotes to support the theme: Addressing healthcare barriers to providing nutrition education in MS care.

| **Key considerations** | **Exemplar quotes** |
| --- | --- |
| **Addressing educational gaps** | **MS-specific PEMs**  *“That was one of the challenges is that all of the information is one size fits all, not MS”* (P07, PlwMS, 7.5 years)  *“I think the biggest thing that came out of that discussion about the challenges was the lack of support and information about diets”* (P14, PlwMS, 6.5 years)  *“I think there are gaps in various areas. I think there should be various resources to be tailored to each cohort”* (P16, Dietitian, 2 years)  *“Patients are hungry for something they can read…, I think people are hungry for information they can depend on”* (P11, Neurologist, 40 years)  *“I have some written handouts that I can talk through with my patients, none MS-specific, most of them are the neuro specific or Parkinson's…, I think the gap for me is not having the MS-specific resources”* (P19, Speech pathologist, 5 years)  *“It's really hard for us to sort of support patients, and it would be good to have an evidence-based educational tool that we could give them”* (P18, Nurse, 10 years) |
|  | **Misinformation and understanding among plwMS**  *“It makes it harder for me to be able to change those misinformation that they have been delving to from Google or from like not reliable resources”* (P13, Dietitian, 1 year)  *“The second challenge I think is just misinformation, so there's so much misinformation about different diets and how they can change MS itself”* (P10, Neurologist, 12 years)  *“Patients don't see how like they know it plays a huge role. They probably don't understand how much it can impact their quality of life or their symptoms”* (P15, Exercise physiologist, 4 years) |
|  | **Non-dietitian HCP knowledge and interest**  *“Neuro {neurologist} not interested and would not contribute to that conversation”* (P14, PlwMS, 6.5 years)  *“I feel I am educating my healthcare professionals about my condition and diet”* (P01, PlwMS, 34 years)  *“I wish I had some sort of you know information to give him”* (P18, Nurse, 10 years)  *“My knowledge is not that great regarding nutrition and usually we don't give advice”* (P17, GP, 18 years)  *“I think there's a lack of interest from health professionals sometimes because eating is something you do anyway, so I think like I could refer you into an infusion clinic because it's like we need you to engage in this thing that you've never done before”* (P03, Nurse, 24.5 years) |
|  | **Dietitian training in MS**  *“My dietitian has been great but she does not know much about {X} diet…, I don’t get support from her on that diet”* (P14, PlwMS, 6.5 years)  *“The dietitian whilst helpful was not trained in specialised MS nutrition”* (P09, PlwMS, 2 years)  *“We don't have a neurology interest group. The closest we have is a disabilities interest groups that's covering a lot of broad, much broader range”* (P05, Dietitian, 0.5 year)  *“A massive barrier for us is a lack of a dietitian knowledgeable about MS in* {X}*…, If there was a little short course or something that dietitians could do specifically about nutritional recommendations for MS, then that would be a beneficial thing”*(P07, Nurse, 10 years)  *“Dietitians needs continual training because things change really quickly”* (P11, Dietitian, 2 years) |
| **Overcoming healthcare systematic barriers** | **Consultation time constraints**  *“Time can be a barrier, I mean time pressure is always a problem in healthcare”* (P10, Neurologist, 12 years)  *“I think we would all agree that the challenge is time. Who has it? There's not enough time in the consultations. There's not enough time for the patients to process the information”* (P01, Nurse, 7 years)  *“I think wanting to have something that could help us with our time factor because we have so much to cover, you know If we had a tool that we could use that was flipped in quite quickly, would be helpful”* (P18, Nurse, 10 years) |
|  | **Limited access to dietitians**  *“When speaking to MS {X} or other healthcare professionals, it doesn’t seem that dietitians are readily available to us’* (P12, PlwMS, 2 years)  *“I think the biggest problem is having a bit of a shortage of dietitians”* (P09, Physiotherapist, 11 years)  *“It's not like there's a particular person that we can refer to who…, from a dietitian standpoint”* (P07, Nurse, 10 years)  *“The access to dietitians is not great, where I am…, so one of the challenges dietitian access”* (P12, Neurologist, 40 years)  *“They might take time to see the patient, if for example, for weight loss. We have patients they wish to see dietitian for weight loss, so this is not urgent”* (P17, GP, 18 years)  *“If you're going to wait to go to a dietitian through the public system, it's a long waiting list as far remember”* (P05, Dietitian, 0.5 year) |
|  | **The cost of dietetic services**  *“NDIS recent changes have made it more difficult to access services including dietitian”* (P06, PlwMS, 10 years)  *“There's a lot of healthcare costs, so people might not necessarily have the funds available to access privately. So I think having more access, in terms of whether it's NDIS funding, or I guess the MS centres”* (P14, Dietitian, 4 years)  *“There is something called the pro bono financial advice network…, and so wouldn't it be cool if there was a network of dietitians who normally charge a gap fee, provide initial consultation, or a one-hour consultation per year”* (P03, Nurse, 24.5 years) |
| **Bridging knowledge evidence gaps** | **Research and guidelines on MS and diet**  *“Evidence-based is important for validation eating a certain diet”* (P05, PlwMS, 1 year)  *“The evidence isn't there, like to really spruik it, the fact that you need to go to alternative, there isn't level A evidence. So, it's suggestive”* (P11, Dietitian, 2 years)  *“I think there's definitely room to grow, but it needs more research”* (P14, Dietitian, 4 years)  *“My frustration is specifically there is no specific nutrition guideline for managing MS…, a practice guideline toolkit for dietitians, especially for new graduates”* (P16, Dietitian, 2 years) |

**Abbreviations.** HCPs: healthcare professionals; GP: general practitioner; MS: multiple sclerosis; NDIS: National Disability Insurance Scheme; PEMs: patient education materials; PlwMS: person living with MS.

**Table 2** Participant quotes to support the theme: Supporting person‑centred nutrition communication.

| **Key considerations** | **Exemplar quotes** |
| --- | --- |
| **Considering plwMS’ readiness and motivation to receive nutrition advice** | *“Also the readiness to accept information is often you know a block as well”* (P02, Psychologist, 3 years)  *“One of the things I usually do in my practice is asking, what the patient wanted to achieve, you know, they might want, they might not want to lose weight”* (P01, Nurse, 7 years)  *“Diet definitely does come up and something I talk to patients about, …so often people will ask”* (P10, Neurologist, 12 years)  *“We speak to people who want to speak to us and about what they want to speak about, but yes, either they directly want to speak about diet, or we're generally opening up the conversation about MS and living well with MS”* (P01, Nurse, 7 years) |
| **Respecting plwMS’ choices and preferences** | *“Every time I mentioned MS diet I was on, I certainly wasn't not pushing it, any discussion around {X} diet was shut down”* (P14, PlwMS, 6.5 years)  *“You want to give them a sense of control and you want to support their choices, and probably don't really feel happy about having to say it's not going to cure your MS”* (P07, Nurse, 10 years)  *“Ultimately, it's their choice if they want to go on an exclusion diet…, you know directing them or redirecting them to the right organisations or reputable sources of information”* (P03, Nurse, 24.5 years)  *“Making sure that we're at least giving people information about consequences as well, but also supporting their choices”* (P05, Dietitian, 0.5 year) |
| **Providing practical and realistic dietary options** | *“I also think the information should be principle information on how you can adapt your diet”* (P07, PlwMS, 7.5 years)  *“Going home with options you can research and going to one place”* (P05, PlwMS, 1 year)  *“I am not keen on strict food choices, would like nutritional alternatives”* (P13, PlwMS, 1 year)  *“Kind of giving her {patient] practical options, alternatives to manage her conditions and also to enjoy food based on her favourite foods”* (P16, Dietitian, 2 years)  *“It's still enjoying having a sustainable diet and enjoying the sometimes treats, not having them every day for every meal, but still having things in moderation”* (P07, Nurse, 10 years)  *“Also outlining from the very start that we have options when it comes to eating and drinking”* (P19, Speech pathologist, 5 years)  *“Just not giving them false hope that ‘oh, great, you're doing this, you can go on for the treatment, because you're on this diet that's going to make you miraculously better’ ”*(P04, Psychologist, 21 years) |

**Abbreviations.** MS: multiple sclerosis; PlwMS: person living with MS.

**Table 3** Participant quotes to support the theme: Fostering equity through accessible and inclusive MS-specific educational resources.

| **Key considerations** | **Exemplar quotes** |
| --- | --- |
| **Including specific nutrition and diet topics** | *“MS X just the basic eat well, everyone should eat the same stuff. Focus on foods that help on inflammation”* (P10, PlwMS, 27 years)  *“People with MS don't want general guidelines. They don't want to be told to follow the Australian healthy eating because it doesn't feel relevant to them”* (P14, Dietitian, 4 years)  *“It's important to have information about those different eating patterns, those different styles of eating, so we can make sure they're at least nutritionally complete if they choose to follow them”* (P05, Dietitian, 0.5 year)  *“I think It would be great to have resources that involve how you can treat or manage individual symptoms with nutrition”* (P01, Nurse, 7 years)  *“Something like a resource, like a pamphlet or brochure about the risk of comorbidities, excess body weight and how this relates to diet”* (P03, Nurse, 24.5 years) |
| **Designing credible educational resources** | *“What's helped with some of that work is that, you know, the research base behind that, and people knowing that there's an evidence base”* (P08, Psychologist, 10 years)  *“You just have it more research-based, so you can have the citations”* (P14, Dietitian, 4 years)  *“That information should simplify the evidence base, not overstate the evidence base, nor dismiss evidence base”* (P12, Neurologist, 40 years) |
| **Ensuring educational resources are easily accessible** | *“…would be a website made for access on the mobile phone and then the computer, and it had different kind of tabs or pages on that website”* (P07, PlwMS, 7.5 years)  *“If you can centralise some of these resources. I was thinking specifically around MS Australia because they house a lot of that information around lifestyle modification…, But what you need to do is make sure that everyone knows that that's where they can go, so it's around promoting it sort of right across the board”* (P03, Nurse, 24.5 years)  *“Maybe you link with an existing organisation like MS plus or MS Australia, because they're well known in the MS space”* (P14, Dietitian, 4 years)  *“If we host them {educational resources} on a website, and then that website link to for example MS Australia…, or it may be that University of {X} wants to host it and the local, the state things can print a flyer with a QR code to your website”* (P12, Neurologist, 40 years) |
| **Providing blended learning educational resources for HCPs** | *“Thinking about education for help dietitians or other professionals, maybe something like CPD webinars would actually be quite helpful…, So maybe it could be to work with an organisation that offers like CPD”* (P14, Dietitian, 4 years)  *“As nurses, we always need to do our professional development kind of hours per year. So that sort of webinars would be really helpful…, so we can look back at our own time and run through”* (P18, Nurse, 10 years)  *“An online webinar is nice, it's always there and whenever I need to look, I just open and look at it”* (P17, GP, 18 years)  *“Like an FAQ style or like having, you know, really common research questions…, More about for clinicians like a summary booklet or sheet about the MS-specific diets, answer evidence to these”* (P14, Dietitian, 4 years)  *“You know run a workshop at the (X} conference, the annual (X} conference or do a training webinar”* (P11, Dietitian, 2 years) |
| **Designing digital, printable PEMs** | *“GPs can send you away with a paper copy”* (P14, PlwMS, 6.5 years)  *“Good for healthcare professionals who are not dietitians to hand us a brochure…, website is a good idea with printable”* (P12, PlwMS, 2 years)  *“I think having different formats for different people will be helpful, so maybe an older audience would want just a paper format they can pick up from their neurologist, but for a younger audience, you want more like alternative, digital”* (P14, Dietitian, 4 years)  *“Links are always great, most of my work is on Zoom, so a link that I can send through an email is helpful, or I can share my screen…, a pamphlet that's linked to a website that's fine, it's on a website”* (P04, Psychologist, 21 years)  *“Like printable resources, I think it would be an online like, an online article that you could then print, you know, becomes in a resource that then is quite tangible to share with patients”* (P01, Nurse, 7 years)  *“A quick pamphlet, brochure, or like a one-page summary, those sort of things are always nice, so like what we usually do is we got like a few websites where we would download like a resource sheet”* (P15, Exercise physiologist, 4 years)  *“A small booklet with practical suggestions, and then maybe you can put a QR code at the page”* (P12, Neurologist, 40 years) |
| **Creating simple, easy-to-understand PEMs** | *“Nothing simple and easy to read, unlike heart health and diabetes which is easier”* (P04, PlwMS, 9 years)  *“I think one pamphlet will not cover all MS and nutrition information in a simple way”* (P03, PlwMS, 0.5 year)  *“We need simple materials because there's no way health literacy is very important, and sometimes people living with MS experience low health literacy”* (P16, Dietitian, 2 years)  *“I think just keeping it simple is always the best way to present the information”* (P10, Neurologist, 12 years)  *“I think even just having a national resource like my stroke journey booklet…, I quite like it, so it uses simple language”* (P19, Speech pathologist, 5 years)  *“Not too much information, I think concise would be important. These patients get overwhelmed, I suppose, by how much we give them”* (P18, Nurse, 10 years) |
| **Including visual aids in PEMs** | *“If you want people with it being able to interpret them and use them, they need to be visual”* (P11, Dietitian, 2 years)  *“Like just visual aids and interactive format could also make the information more engaging and easier to understand”* (P13, Dietitian, 1 year)  *“Visuals have to be the right visuals…, for example, you can't put a visual when you're saying don’t eat that on top of it”* (P16, Dietitian, 2 years)  *“We are in a sort of an area where literacy is not always great here as well. So something that even pictures and things would be helpful, so an easy visual tool”* (P18, Nurse, 10 years) |
| **Designing PEMs with clear visual formatting** | *“The resources should have a bright background and contrasting colours large font size and separation of different headings”* (P01, PlwMS, 34 years  *“You know what it would be using all those disability guides…, when you're creating these resources, that talks all about font size, spacing, even font style, all of that business amount of content per page, heading, subheading”* (P11, Dietitian, 2 years)  *“The best way to present the information so like not too much colour, and consistent scheme professional”* (P10, Neurologist, 12 years) |
| **Offering audio-format PEMs** | *“It should also be accessible, so for people who have visual impairment, there should be an audio version”* (P06, PlwMS, 10 years)  *“We could package the same message, but in a couple of different ways, because some people are going to be more visual, some people will be more auditory, you know, and they might just hear it”* (P04, Psychologist, 21 years) |
| **Representing diverse backgrounds and cultures in PEMs** | *“The resources can be in different languages for accessibility or maybe something in the future”* (P01, PlwMS, 34 years)  *“Infographics should represent diversity, you know for example MS impacts men too not only women”* (P03, PlwMS, 0.5 year)  *“I think any images should be diverse in their representation of all people or foods or cultures…, you know you want something that's really people could relate to”* (P01, Nurse, 7 years)  *“Culture is very important…, with these different foods if we want to create a list of foods”* (P16, Dietitian, 2 years) |

**Abbreviations.** CPD: continuing professional development; HCPs: healthcare professionals; GP: general practitioner; MS: multiple sclerosis; PEMs: patient education materials; PlwMS: person living with MS; QR: code generator.

**Table 4** Participant quotes to support the theme: The integral role of the dietitian in MS care.

| **Key considerations** | **Exemplar quotes** |
| --- | --- |
| **Accessing to individualised nutrition advice** | *“A number of sessions with a dietitian for one on one advice would be great”* (P04, PlwMS, 9 years)  *“It would be good for you to see a dietitian to get a food plan from a dietitian”* (P10, PlwMS, 27 years)  *“If the person is deeply interested in diets, they should be working one on one with dietitians to get that deep understanding”* (P04, Psychologist, 21 years)  *“We all know that when a person living with MS, research suggests that may have co-morbidities and also the actual depression and anxiety because of the MS conditions that they live with…, I think they do need a dietitian”* (P16, Dietitian, 2 years) |
| **Supporting sustained dietary behaviour change** | *“We have the information but what do we do with that then”* (P05, PlwMS, 1 year)  *“With dietary behaviour changes, I know we're not psychologists, but sometimes we tend to be in that category in a way, so we sit there and sort of listen to the patient and their frustration and their clinical symptoms”* (P16, Dietitian, 2 years)  *“The reason they {plwMS} lose weight may not be the surgery so much of the fact that they're seeing a dietitian regularly and a psychologist,* *and they're being supported”* (P12, Neurologist, 40 years)  *“I suppose, educating patients like MS is a lifetime disease, and what achievable in the short term and what's achievable in the long-term regarding diet and support from dietitians”* (P07, Nurse, 10 years) |
| **Promoting interdisciplinary collaboration through dietitian referrals** | *“If they're talking to me because they're more interested and they want more information, and I know that's out of my scope, certainly I would give them a referral”* (P04, Psychologist, 21 years)  *“A quick screening tool, so we can identify where people are coming from, so we know exactly as best possible to help them and understand their referral and treatment pathway”* (P01, Nurse, 7 years)  *“I reckon there should be resources for GPs and neurologists to guide referrals to a dietitian”* (P16, Dietitian, 2 years)  *“It would be good or valuable to have a platform…, to find an MS dietitian near me that I can refer to”* (P15, Exercise physiologist, 4 years) |
| **Raising awareness about the role of dietitians MS care** | *“I do have the impression that dietitian is not being taken seriously by the system”* (P09, PlwMS, 2 years)  *“I don't think GPs in general, they have much knowledge about they need to refer to dietitian if they have MS patient”* (P17, GP, 18 years)  *“The neurologists, I don't think really appreciate the role you know, allied health professionals play particularly…, you know having that holistic approach and just having that knowledge and awareness of dietitians maybe raised before in”* (P06, Dietitian, 3 years)  *“People usually ask the neurologists, or maybe even their GP. So having some more information for helping healthcare professionals about what a dietitian can help with in the MS space would be helpful”* (P14, Dietitian, 4 years) |

**Abbreviations.** GP: general practitioner; MS: multiple sclerosis; PlwMS: person living with M.

Supporting Information 5: Supporting quotes illustrating the key themes (Phase 3)

**Table 1** Participant quotes to support the theme: Clear, targeted messaging as a driver of action.

| **HCPs** | ***What participants liked***  *“I think that the helpful thing about the content is, it tells you why you're doing this? I think you've made it clear that the role of nutrition”* (P09, Neurologist, 12 years)  *“Like it's printed it out {booklet}, and yeah, like it's simple enough to be simple, but it's got enough information, and that it's actually really useful and helpful”* (P11, Nurse, 10 years)  *“I think it's {screening tool} simple. That could be done quite quickly in the purpose of the consult…, I even think if the person wasn't really ready to see a dietitian in my hospital. I could add stuff like that tacked onto the GP letter”* (P10, Nurse, 27 years)  *“I like it, the patient section, because I can see how, when the patient is usually in the clinic. They have so many questions detailed question, and they can't ask it through the clinic time”* (P15, GP, 11 years)  *“Oh, wow! That comes straight up with all the dietitians in our area…, I've never seen anything like it actually. Love it absolutely love it. You've covered everything from funding to diet and dietitians”* (P16, Nurse, 10 years)  *“I like the practice points. So just like key summary, you know, encourage hydration to prevent constipation…, it's kind of got everything that you want in there {booklet}”* (P17, Dietitian, 5 years)  *“I think they seem really good putting in brackets what the actual like the Wahls diet is because I couldn't remember exactly which one that was, you know, like what they omit”* (P08, Dietitian, 15 years)  ***Suggestions for improvement***  *“It might be useful to have a little bit more details around what each of them {sections on the websites} has inside it…, just to know what is in those in yeah, in those pages”* (P13, Psychologist, 4 years)  *“My experience with specialist physicians and general practitioners is that they really like so something that very clear and very explicit that they can just follow”* (P14, Exercise physiologist, 1 year)  *“Just simple things like that, so that we can be very clear because if I'm sitting with the patient, I can't read this to them. So part of the job I have to do is read dyslipidaemia. Remember what that is, and then say, okay. The next question is about like your cholesterol”* (P01, Nurse, 8 years) |
| --- | --- |
| **PlwMS** | ***What participants liked***  *“I do believe that gives information and is presented in a way that is understandable”* (P12, PlwMS, 2.5 years)  *“It is very clear for people whose English might not be really good”* (P08, PlwMS, 1.5 years)  *“The suggestions there seem really practical…, There wasn't a lot of new information in there, but it was ensuring that I hadn't gone down the wrong track”* (P01, PlwMS, 10.5 years)  *“There was one thing that stood out that I didn't know. Calcium, the medications can take the calcium, because that's happened to me…, I've never seen written about MS in all the all my journey. Good that people are picking up on it”* (P03, PlwMS, 23.5 years)  *“Oh, love absolutely love QR code for anybody that teaches friendly cooking”* (P05, PlwMS, 9 years)  *“Most of the resources these days, they're more about explaining why this happens and more content, heavy and theoretical. But this resource is more like tips and things. People can actually implement like small changes they can do in their lives”* (P14, PlwMS, 2 years)  *“I've never seen a resource that has clearly specified those things and given instructions on how you can read food labels better”* (P14, PlwMS, 2 years)  ***Suggestions for improvement***  *“If people have been having white bread, while where you have the back of the label that you could explain,….Why it's better to use, or, you know, use a multigrain rather than this, and this is why and these are the effects that could cause you, and then it kind of gives them a reason to back up why you're suggesting these foods over those foods”* (P06, PlwMS, 3 years)  *“Obviously eating whole grains and dairy and stuff, It's helpful for everyone. But this fact sheet is meant for a specific cohort. So having that link with MS is obviously better”* (P14, PlwMS, 2 years)  *“Maybe a first sentence could be what metabolic health is. Yeah, in really plain English”* (P07, PlwMS, 1.5 years)  *“A lot of this was on here in the fatigue section I did find had already come up elsewhere, so I don't know if it's repeating the same”* (P09, PlwMS, 5 years)  *“So that one is very, very wordy. I actually probably just switched off immediately when I saw lots of words”* (P10, PlwMS, 1 year) |

**Abbreviations.** HCPs: healthcare professionals; GP: general practitioner; MS: multiple sclerosis; PlwMS: person living with MS; QR: code generator.

**Table 2** Participant quotes to support the theme: Visually engaging and informative design.

| **HCPs** | ***What participants liked***  *“I just clicked on the link {website}, and then I just followed. I went to the clinician section first, and then I went to the patient section and I just clicked on the links and have a look at the different fact sheets. I just navigated through it, as it was quite intuitive”* (P18, Neurologist, 20 years)  *“It's {website} user friendly, which is probably the main thing that I want…, I think that's like always going to be the number one reason that I will keep using a tool is actually being able to navigate it and just the simplicity of it”* (P07, Exercise physiologist, 5 years)  *“This kind of tool {website} is informative, simple, and navigating from one page to another one is easy and not complicated. So easy, visually”* (P04, GP, 15 years)  *“I like how you've got it {website} in different sections, like, it's quite user friendly to actually just sort of link into…., I love the fact that you put on page 8, you put things in boxes, I do like that in each section, I do like that makes it a lot easier to glance at to see what I'm looking for”* (P16, Nurse, 10 years)  *“I think it {website} sets everything up in sections which is always positive rather than just all, one after another. You know it's I guess chunking, I think, is a word that I've heard of. That is a good thing”* (P08, Dietitian, 15 years)  *“I have a gut feeling that when these {fact sheets} become available, I'll pick print on everything and actually create our own little booklets that have every bit in it”* (P10, Nurse, 27 years)  ***Suggestions for improvement***  *“I'm trying to format this just to look a bit more professional for me. This looks a bit. I'm not going to say the word childish, but it just looks a bit bright, and I think some colours are unnecessary, so trying to make it {booklet} look a bit more formal”* (P09, Neurologist, 12 years)  *“You know, click here for meal ideas or click here for shopping list, like. So if people want to just go to that because scrolling down can be fatiguing.... So I think it makes it more accessible for people if they have some more grouping so they can click and get to”* (P13, Psychologist, 4 years)  *“Small thing I would prefer it if the text was justified…, It looks like the alignment has gone off a bit”* (P12, Occupational therapist, 27 years)  *“Make it easy to read how to use in practice…, potentially using like italics or bold”* (P08, Dietitian, 15 years)  *“If we look at that fact sheet, for example, photocopying the top blue that's dark blue with red…, but try to keep most of the sheet in white”* (P05, Psychologist, 23 years) |
| --- | --- |
| **PlwMS** | ***What participants liked***  *“I like the way how it's done like with the little pieces of a little table with the dot points”* (P12, PlwMS, 2.5 years)  *“The pictures of the products are really good..., you could send someone, you know, a support worker, or whatever down the road and say, buy these ones”* (P01, PlwMS, 10.5 years)  *“Found the boxes a lot easier than say, perhaps further up the page where it's a big thing of text”* (P09, PlwMS, 1 year)  *“it's a good visual, visualize thing, because when you go shopping, you can actually see it, which ones to get., yeah, so it makes it a lot easier”* (P02, PlwMS, 24 years)  *“I like it because it explains it, and you again. You've got your little boxes when it goes from one to the other”* (P03, PlwMS, 23.5 years)  *“I thought they were good, that they were broken into different fact sheets for different topics”* (P07, PlwMS, 1.5 years)  ***Suggestions for improvement***  *“If we can remove the white border or yeah around all the images, because the ones that are down further, like the apple and kiwi fruit and all that looks really good where there's no white border”* (P06, PlwMS, 3 years)  *“It could be improved, maybe make it a little bit nicer just a little bit more appealing”* (P08, PlwMS, 1.5 years)  *“But trying to use visual bits and then put key points”* (P04, PlwMS, 10 years)  *“I think that was actually one of my feedback that the blue background on the heading. It's hard to read the text with red, but you've already changed it. So that's brilliant”* (P14, PlwMS, 2 years) |

**Abbreviations.** HCPs: healthcare professionals; GP: general practitioner; MS: multiple sclerosis; PlwMS: person living with MS.

**Table 3** Participant quotes to support the theme: Lived experiences informing choices.

| **HCPs** | ***What participants liked***  *“I think you've done it really, really well, because so many consumers will have heard around about some of these specific diets for MS and you've just presented in a very non-judgmental but gently directive way”* (P05, Psychologist, 23 years)  *“The aspects that I liked were the one, the fact that the recommendations weren't very dogmatic. So there's a lot of options for patients. I guess, because different people have different dietary preferences”* (P18, Neurologist, 20 years)  *“You can choose from the summary booklet what you want to, you know, follow up with the patient or client and it does cover all the areas. Yeah, I do think they are flexible…, but having all those options would be great, and then more likely, depending on where you work”* (P08, Dietitian, 15 years)  *“It {website} just gives you an option to look at…, You can take all of it, or you can take one bit of it depending on what's needed and who you're sharing the resources”* (P06, Speech pathologist, 5 years)  ***Suggestions for improvement***  *“And then just acknowledging the emotional aspect of food…, so acknowledging food is really personal, It's cultural, you may have dietary restrictions, you may have cultural restrictions”* (P01, Nurse, 8 years)  *“About the whole grains. Yes, but I still do not eat brown rice…, it's not a staple for most Western, you know for white Australians.., that's not going to work”* (P02, Dietitian, 1 year)  *“The only thing I might suggest is just because salmon is featured in 3 of those pictures, maybe just finding another one, perhaps with a different protein or a vegetarian option for some of perhaps the dietary guidance”* (P06, Speech pathologist, 5 years) |
| --- | --- |
| **PlwMS** | ***What participants liked***  *“I like the swap for options that are in there. So yeah, it's not saying no to all fast and fried, yeah, to all foods, but just swapping out bit by bit that helps with habit forming”* (P07, PlwMS, 1.5 years)  *“I think that you obviously made a point of including options for vegetarian people. You know, and I think that everyone that has dietary requirements”* (P08, PlwMS, 1.5 years)  *“I like the fact that, you know, like all these other things like takeaway food, you know, like Mcdonald's, or whatever. When you're feeling hungry you can swap it”* (P02, PlwMS, 24 years)  *“You kind of give guidance on what people could choose and then they can fit it into their life”* (P03, PlwMS, 23.5 years)  *“I thought that was really good sort of a very much, this is the information, what you choose to do is up to you, but if you do go down this path. you know. Here's a suggestion of to make sure you are getting those nutrients your body…, It's not sort of saying you have to”* (P11, PlwMS, 0.25 year)  ***Suggestions for improvement***  *“It's very well laid out. I mean, maybe if you just put something in there and say…, you know, please adjust to yours”* (P13, PlwMS, 32.5 years)  *“Just recognise the diversity now that you know, resides in Australia, it's not all Western, you know. You'll have some cultures that eat other types of foods”* (P08, PlwMS, 1.5 years)  *“We could modify the information, you know, to suit those who are cooking or buying from outside…, you could just have a section like the snacks, like, just dining out considerations”* (P04, PlwMS, 10 years)  *“I noticed in all of this you wrote about vegetarian or vegan, but you didn't write anything about gluten free, or people with allergies”* (P05, PlwMS, 9 years) |

**Abbreviations.** HCPs: healthcare professionals; MS: multiple sclerosis; PlwMS: person living with MS.

**Table 4** Participant quotes to support the theme: Building relationships through trust, credibility and connection.

| **HCPs** | ***What participants liked***  *“What I liked most about that, it seemed to be mostly evidence based, which is good”* (P18, Neurologist, 20 years)  *“At least, this is {booklet} coming from somewhere credible. So I think that's really important”* (P16, Nurse, 10 years)  *“I think when people raise dietary concerns with me, it will be really nice to be able to send them new up-to-date MS specific information about dietary factors”* (P08, Dietitian, 15 years)  *“Knowing that I can go to here {website}, and even as a clinician like I can, I feel confident, feel comfortable, giving something that I know is evidence based”* (P07, Exercise physiologist, 5 years)  ***Suggestions for improvement***  *“It adds a bit of additional weight to it. I guess that the information that is in there is has not only been accredited by dietitians, but it's also by those who specialise in MS…., you know, once you finalised it. If you get, because then they'll {HCPs} also refer to it”* (P03, Nurse, 24.5 years)  *“Includes a summary, or a top line ‘review of current best practice information that through the University of {X}, has been interviewed and researched with relevant Australian MS health professionals’”* (P01, Nurse, 8 years)  *“I'm wondering what that is that organisation? I've not heard of it before…, I think if I'm going in there as a healthcare professional and I'm wanting to get evidence-based information that I'm gonna pass on to my clients I need to know where it's coming from”* (P12, Occupational therapist, 27 years)  *“I wonder for fatigue, make under fatigue, like, maybe consider occupational therapy input”* (P11, Nurse, 10 years)  *“Whether or not you simply refer them back to this page…,I think it's just being conscious that not everyone, even if there's {dietetic services} one in their local region, they {plwMS} can't afford it, or that you know whether there's something to remind them that the health professional, that there are plans and ways, you know, to get the limited number of free Medicare”* (P02, Dietitian, 1 year) |
| --- | --- |
| **PlwMS** | ***What participants liked***  *“Underneath, it just makes it credible, doesn't it like it's something that's being reviewed by dietitians”* (P06, PlwMS, 3 years)  *“Last updated April 25. That's good I think it's good because it shows you how update the information…, If it was 10 years ago it wouldn't be in date. Makes a good quality”* (P02, PlwMS, 24 years)  *“The way it's presented it seems to be that someone who does know about MS has written this. It's not someone who has no idea”* (P12, PlwMS, 2.5 years)  *“Particularly one that I like, and one that I didn't know early on in the piece was flagging up that you consult your doctor with the calcium supplement, because you don't want too much”* (P09, PlwMS, 5 years)  ***Suggestions for improvement***  *“I would also mention the NDIS and also mention like, for example, exercise physiologist that you do get support in that”* (P12, PlwMS, 2.5 years)  *“I think it's important to have the link to MS Australia, so that any questions can be directed to MS Australia”* (P07, PlwMS, 1.5 years)  *“I think you probably just need this one to, you know, if you're suffering these particular issues, you know, you need to discuss it with your medical practitioner. Yeah, link it back to them”* (P10, PlwMS, 1 year)  *“If you put also people with lived experience of MS…, It might make people more accepting of it…., and they might go ‘Oh, this isn't just something that some dietitians done. It's something that people with this condition have worked on’..., co-designed with support of the MS community, or something, I think so, because people will be a bit paved”* (P13, PlwMS, 32.5 years) |

**Abbreviations.** HCPs: healthcare professionals; GP: general practitioner; MS: multiple sclerosis; NDIS: National Disability Insurance Scheme; PlwMS: person living with MS.
